# Tractography-based navigated TMS language mapping protocol

**DOI:** 10.3389/fonc.2022.1008442

**Published:** 2022-12-09

**Authors:** Klara Reisch, Franziska Böttcher, Mehmet S. Tuncer, Heike Schneider, Peter Vajkoczy, Thomas Picht, Lucius S. Fekonja

**Affiliations:** ^1^ Image Guidance Lab, Department of Neurosurgery, Charité – University Hospital, Berlin, Germany; ^2^ Cluster of Excellence: “Matters of Activity. Image Space Material”, Humboldt University, Berlin, Germany

**Keywords:** language mapping, tractography, glioma, preoperative planning, diffusion magnetic resonance imaging, transcranial magnetic stimulation

## Abstract

**Introduction:**

This study explores the feasibility of implementing a tractography-based navigated transcranial magnetic stimulation (nTMS) language mapping protocol targeting cortical terminations of the arcuate fasciculus (AF). We compared the results and distribution of errors from the new protocol to an established perisylvian nTMS protocol that stimulated without any specific targeting over the entire perisylvian cortex.

**Methods:**

Sixty right-handed patients with language-eloquent brain tumors were examined in this study with one half of the cohort receiving the tractographybased protocol and the other half receiving the perisylvian protocol. Probabilistic tractography using MRtrix3 was performed for patients in the tractography-based group to identify the AF’s cortical endpoints. nTMS mappings were performed and resulting language errors were classified into five psycholinguistic groups.

**Results:**

Tractography and nTMS were successfully performed in all patients. The tractogram-based group showed a significantly higher median overall ER than the perisylvian group (3.8% vs. 2.9% p <.05). The median ER without hesitation errors in the tractogram-based group was also significantly higher than the perisylvian group (2.0% vs. 1.4%, p <.05). The ERs by error type showed no significant differences between protocols except in the no response ER, with a higher median ER in the tractogram-based group (0.4% vs. 0%, p <.05). Analysis of ERs based on the Corina cortical parcellation system showed especially high nTMS ERs over the posterior middle temporal gyrus (pMTG) in the perisylvian protocol and high ERs over the middle and ventral postcentral gyrus (vPoG), the opercular inferior frontal gyrus (opIFG) and the ventral precentral gyrus (vPrG) in the tractography-based protocol.

**Discussion:**

By considering the white matter anatomy and performing nTMS on the cortical endpoints of the AF, the efficacy of nTMS in disrupting patients’ object naming abilities was increased. The newly introduced method showed proof of concept and resulted in AF-specific ERs and noninvasive cortical language maps, which could be applied to additional fiber bundles related to the language network in future nTMS studies.

## 1 Introduction

Patients with brain tumors in language-related areas are at a high-risk for developing post-operative language deficits (1). Thus, a major objective in neurosurgical planning is determining functional roles of anatomical areas and establishing reliable tools to do so (2). Transcranial magnetic stimulation (TMS) is a method of neurostimulation where an electromagnetic coil is placed on a subject’s scalp and through the induction of electromagnetic currents, results in the depolarization of underlying neurons (3). The stimulation, when paired with a language task, can elicit TMS-induced language errors, in an attempt to assess the functional significance of cortical areas based on error type and location (4). The use of TMS for neurosurgical language mappings has provided a valuable, feasible and non-invasive method to identify language-relevant cortical areas used in preoperative planning assessments (5). However, many considerable challenges concerning its reliability have been reported, especially with nTMS errors showing low overall specificity rates (3, 4, 6, 7).

Initially, TMS language mappings utilized external cranial landmarks as stimulation points (8). With the incorporation of navigated TMS (nTMS) and the registration of the electromagnetic coil with the subject’s structural MRI, specific brain regions could be targeted in real-time (9). Protocols for nTMS language mapping have focused predominantly on stimulations based on cortical regions or stimulating generally across a patient’s cortex to identify language-relevant areas (4, 10). Yet, this approach overlooks the architectural differences of the patient’s white matter, which is especially relevant in patients with tumor-induced distortions.

Current understanding of language processing is strongly based on the structural and functional interconnectivity of complex neuronal networks through various white matter tracts (11, 12). Though the functional significance of these connections remains widely unknown, contemporary models have improved our understanding of network interactivity in relation to function. The current dual-stream model of cerebral language proposes a ventral stream, involved in processing sound-to-meaning encoding as well as a dorsal stream, involved in processing sound to articulation (13–15). The bilaterally-organized ventral stream is involved in semantic processing and comprises the middle longitudinal fasciculus (MdLF), the inferior fronto-occipital fasciculus (IFOF), the inferior longitudinal fasciculus (ILF), the extreme capsule, and the uncinate fasciculus (UF) (13). The dorsal stream mainly comprises the arcuate fasciculus (AF), largely viewed as the most important tract in language processing (16) with significance in the preservation of speech production, repetition, naming, and fluency (17). Impairments to the AF during resection procedures have shown to be relevant in lasting post-operative aphasia, especially around the temporo-parietal-occipital junction (18, 19), making the AF an important subject in language studies.

Diffusion weighted imaging (DWI) methods allow for the production of individual fiber tract maps with their corresponding cortical terminations or nodes (20). The feasibility of performing nTMS-based mappings with diffusion tensor imaging (DTI) fiber tracking has been shown in tracts related to motor function, like the corticospinal tract (CST), where nTMS has demonstrated robust results (21, 22). Considering language function, nTMS-based DTI fiber tracking in combination with anatomically-based regions of interests (ROIs) have demonstrated increased reliability and better reconstruction of language networks when compared to conventional DTI (5). Correlations have been shown between AF endpoints and nTMS-induced language errors (23, 24) and a recent study investigating the functional changes in glioma patients used nTMS positive sites for connectome analysis (25).

The aforementioned studies demonstrate the use of nTMS errors as seeding sites for tractography and diffusion studies. Conversely, tractography endpoints could be implemented as stimulation points in nTMS mappings to better assess the relationship between cortical endpoints of fiber bundles and their effect on language function. In this study, we explore the feasibility of implementing a nTMS language mapping protocol by using AF endpoints as stimulation targets, tracked with constrained spherical deconvolution (CSD)-based probabilistic tractography within the MRtrix3 framework (26). nTMS based on individual fiber tract reconstructions allows for a greater standardization of stimulation target points and an acknowledgement of interindividual differences in white matter, especially when affected by displacing tumors or edema. We hypothesize that our approach increases the error rates (ERs) during nTMS stimulation and shows a different distribution of nTMS errors when compared with a protocol stimulating over the entire perisylvian cortex. This establishes the basis for a tractogram-based nTMS language mapping protocol.

## 2 Methods

### 2.1 Patients

Sixty patients with brain tumors in language function-related areas were prospectively included in this study for pre-operative nTMS. Handedness was determined using the Edinburgh handedness inventory (27). Patients were eligible to participate if they were right-handed, 18 years of age or older, and had a tumor in their left hemisphere. Additionally, all patients received a pre-operative Aachen Aphasia Test (AAT) (28) for assessment of language impairment. Exclusion criteria for the study included multifocal/multicentric studies, multiple tumors, frequent generalized seizures (more than one per week), and general TMS exclusion criteria (i.e. pacemaker, pregnancy, cochlear implant, intracranial clips, Ménières disease). Patients with aphasia too severe to complete the object naming task were excluded from the study.

### 2.2 Ethics approval

This study was approved by the Charité’s Ethics Committee (EA1/005/20) and was performed in accordance with the Declaration of Helsinki. Patients were supplied with a written informed-consent form as well as information on the study and the language mapping procedure prior to their nTMS session.

### 2.3 Neurological assessments

Pre-operative assessments of aphasia were performed using the AAT battery (28), which was adapted into the Berlin Aphasia Score (BAS), developed by physicians at the Charité University Hospital, Berlin and classified patients into a 4-grade system: 0 = no aphasia (≥ 90% AAT Score), 1 = mild aphasia (75 – 89% AAT Score), 2 = moderate aphasia (55 – 74% AAT Score) and 3 = severe aphasia (< 55% AAT Score) (2, 4, 29, 30). AAT sub-tests included: 1. The Token Test (max. 50 points), used to test comprehension and cognitive performance (Ex.: 10 tokens are placed on colored circles and squares. The researcher instructs: “before pointing to the red circle, remove the yellow square”); 2. Naming, in which patients were prompted to name objects, composite nouns, colors, and situations; 3. Repetition, where patients were asked to repeat words or phrases spoken by the researcher, to test for speech production deficits; and 4. Speech comprehension for spoken language and written language, to test for auditory and reading comprehension for words and sentences. Each response was graded from 0-3 for a maximum score of 350 points (28).

Cognitive deficits were additionally assessed pre-operatively using the Demenz-Detection Test (DemTect) (31) a`nd categorized into 3 scores (1 = age-appropriate cognitive abilities, 2 = mild cognitive impairment, 3 = severe cognitive impairment). The AAT was also performed 3-7 days post-operatively to assess for new deficits directly linked to surgery.

### 2.4 MRI acquisition

T1-weighted magnetic resonance imaging (MRI) Data were acquired on a Siemens Skyra 3T scanner (Erlangen, Germany) equipped with a 32-channel receiver head coil at the Charité University Hospital’s Department of Neuroradiology. These data consisted of a T1-weighted structural (TR/TE/TI 2300/2.32/900 m s, 9° flip angle, 256 x 256 matrix, 1 mm isotropic voxels, 192 slices, acquisition time: 5 min) and a single shell dMRI acquisition (TR/TE 7500/95m s, 2 x 2 x 2 mm 3 voxels, 128 x 128 matrix, 60 slices, 3 b 0 volumes), acquired at b = 1000 s/mm2 with 30 gradient orientations, for a total acquisition time of 12 minutes.

### 2.5 Preprocessing of MRI data

To stimulate specific cortical endpoints of white matter tracts, tractography was performed for *in vivo* reconstruction and visualization of the AF (32). The preprocessing of diffusion magnetic resonance imaging (dMRI) data was done using MRtrix3 (26), FMRIB Software Library (FSL) (33), and advanced normalization tools (ANTs) (34) with the following steps as described in (35): denoising (36), removal of Gibbs ringing artifacts (37), correction of subject motion (38), eddy-current correction (39) and susceptibility-induced distortions (40) in FSL (33) and further bias field correction with ANTs. dMRI data sets were visually inspected for artifacts and excessive motion (>10%). No patient data needed to be excluded. Before computing fiber orientation distribution functions (fODFs), data was up sampled to an isotropic voxel size of 1.3 mm to improve anatomical contrast and downstream tractography results using MRtrix3 (26, 35, 41).

### 2.6 Tractography

**Figure 1:**
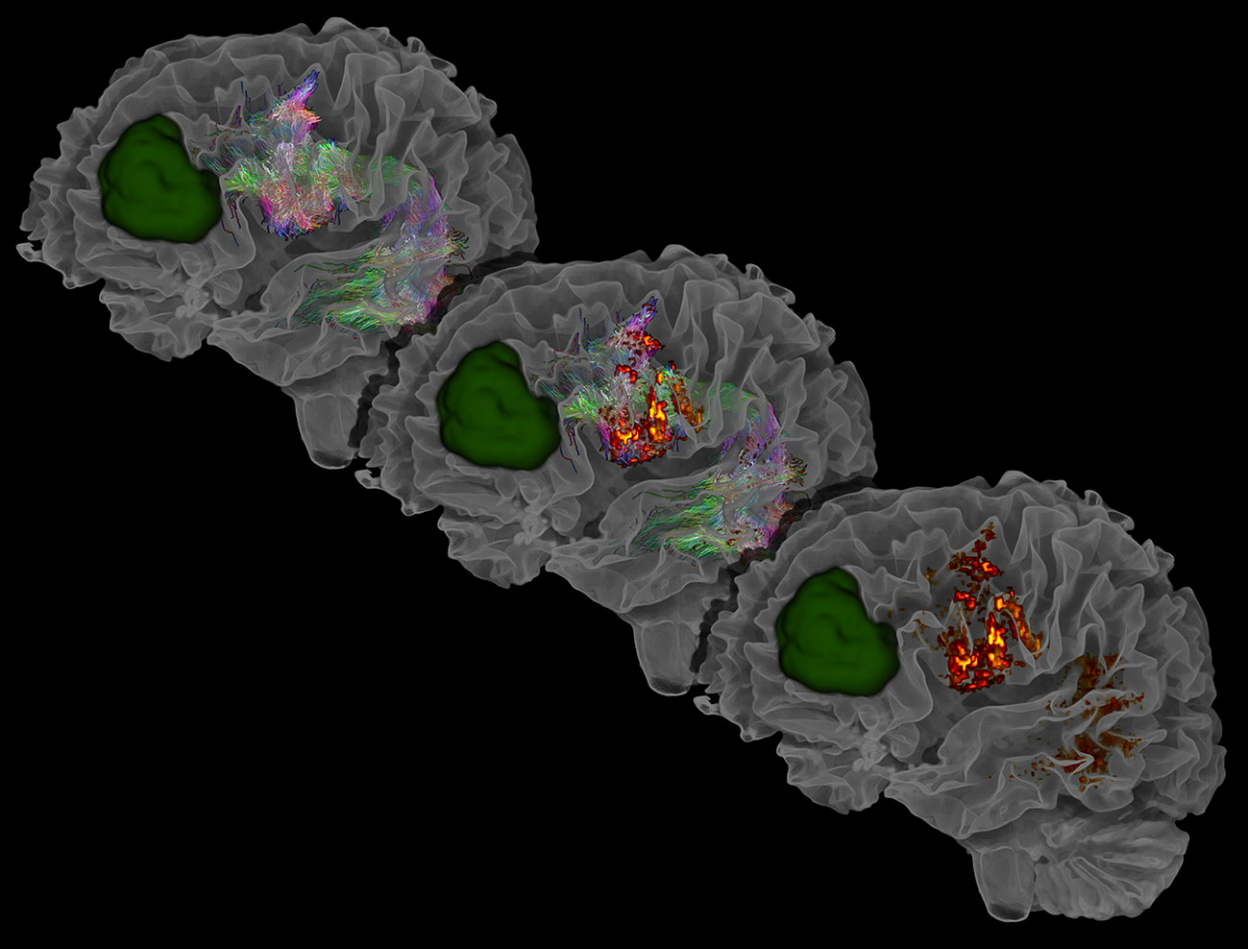
Exemplary visualization of the AF, the AF’s corresponding segmented endpoints, and segmented tumor lesion (green), segmented using ITK-SNAP (42). Visualized are three sequential stages: the initial tractogram (top left), the tractogram with its cortical endpoints (middle), and the resulting cortical endpoints of the tractogram (bottom right). The AF is visible through the transparency setting of the white matter mask. In the frontal region, streamlines can be seen running anterior-posterior (green), showing proximity to the tumor and emphasizing the importance of preoperative mapping. Laterally and posterior to the tumor, reddish streamlines indicating left-right directions can be observed ending in the left cortex.

Tractography was performed using the probabilistic second order integration over fiber orientation distributions (iFOD2) algorithm (**Figure 1**) within the MRtrix3 software framework, which has shown to improve anatomical plausibility compared with deterministic tractography algorithms (20, 26). Default n = 5000 streamlines were selected with an FOD-cutoff value of 0.16 and a minimum streamlines length of 50 mm (35). All ROIs were defined in a standardized fashion (41, 43). The seeding ROIs were set in the coronal view underneath the central sulcus, superior to the circular sulcus of the insula. The second, inclusion ROIs were placed in the temporal lobe in the axial view at the level of the posterior superior temporal gyrus (pSTG). All tracts were visually inspected for anatomical plausibility and spurious streamlines. Exclusion ROIs were included if manual AF editing was necessary due to tumor-related anatomical changes.

### 2.7 nTMS

#### 2.7.1 nTMS language mapping protocol

For the mappings, we used the nTMS eXimia NBS, Nexstim NBS 4.3 and NexSpeech modules (Nexstim, Helsinki, Finland). We uploaded the T1 images with the tractogram-based cortical endpoints to the Nexstim machine and visualized them with a peeling depth of 22.5mm. We performed co-registrations of the patients’ heads with the T1 images using cranial landmarks prior to the nTMS mappings, allowing a maximum stereotactic error of 2.5mm. Each patient’s resting motor threshold (RMT) was measured over the left primary motor cortex for the first dorsal interosseus (FDI) muscle of the contralateral hand. We performed baseline testing in three rounds, each with 80 black-and-white objects that patients were asked to name without leading articles or a lead-in phrase at an inter-picture interval (IPI) of 2500 ms. Objects that patients incorrectly named in the baseline resulted in a removal of these objects from the remainder of the mapping (10).

#### 2.7.2 nTMS stimulation protocols

Half (n=30) of the patients in this study received the new tractography-based protocol while the other half (n=30) received the perisylvian protocol (4). Patients were allocated chronologically, meaning that the first 30 patients were assigned to perisylvian protocol, and the other 30 patients to tractography-based protocol. For the tractogram-based protocol, we used a script (44), which enabled extraction of the AF’s cortical endpoints and visualization of the endpoints. The script includes steps on preprocessing, mapping the cortical endpoints, mapping the endpoints onto the T1 image, and exporting of the T1 file containing the cortical endpoints into the Nexstim system. Transformation to the T1 image was done using the MRtrix3 framework (26). The transformed T1 image with the corresponding endpoints was uploaded into Brainlab using Karawun (45) in order to re-import them into Brainlab at the conclusion of the mapping.> The image was uploaded into the Nexstim TMS system and AF endpoints were visualized. The endpoints were shown as white points projected in the T1 image after the transformation. The endpoints were often grouped in clusters and were shown as white spots on the T1 image. Through visual inspection and by measuring the intensity values of the exported tractogram endings map, the areas of highest endpoints density were selected as stimulation points. On both hemispheres, three frontal cortical points and two temporal-parietal targets of the AF were defined using the crosshair tool in Nexstim and marked with stimulation markers. These markers were then stimulated during the TMS stimulation part of the procedure.> During the stimulation component of the mapping, each of the markers was stimulated a total of 10 times before moving to the next stimulation target.

The other group (n=30) received the perisylvian nTMS mapping protocol, in which the left and right perisylvian cortices were stimulated without targeted stimulation. The T1 image was uploaded into Nexstim and cortical stimulation markers were placed 5 to 10 mm apart, with a total of 80-120 areas per hemisphere. Each area was stimulated once before moving onto the next. Altogether, each area was stimulated three times non-consecutively (4). The patients in the perisylvian protocol-based group did not receive tractography prior to their language mappings. Stimulation parameters for both protocols were set to an IPI of 2500 ms and a picture-to-trigger interval (PTI) of 0ms. Patients were instructed to name objects under stimulation using 110% of the RMT intensity (4) and a frequency of 5 Hz lasting for 5 stimuli (for a total of 1 second per stimulation). The minimum E-field intensity used during mappings was 60 V/m and we sought to keep the intensity during mappings between 60-100 V/m. To maintain a higher intensity, the nTMS coil was oriented perpendicular to the sulcus (10 ***).***

### 2.8 nTMS speech error analysis

Coordinating nTMS stimulation with an object naming task leads to nTMS-related errors (3). The nTMS mapping was recorded using the NexSpeech module (Nexstim Oyj 2018). Within this module, the location of nTMS errors as well as the error types were categorized according to previous studies (4, 46). Errors were classified into five categories: 1. semantic paraphasias, in which the word uttered had a different meaning (‘cat’ instead of ‘dog’); 2. phonological paraphasias, or errors in the sound and pronunciation of the word (‘gog’ instead of ‘dog’); 3. performance errors, including stuttering, slurring, and false articulation; 4. No-response errors, in which there was no-response or a delay lasting longer than the length of stimulation; and 5. hesitations, which was quantified as a prolonged delay not extending beyond the length of stimulation. Errors were classified by two independent and trained researchers with extensive experience in nTMS language mappings.

The evaluation of the results was not done in a blinded fashion. The discrepancies were then compared against each other to reduce the subjectivity of mappings.

Each nTMS error was color-coded and visualized on the patient’s MRI. To identify the cortical areas associated with each nTMS error, a gyral anatomy-based parcellation system (47) was utilized and overlayed onto the T1 image using a cortical brain map, as in previous nTMS language studies (4, 48). This overlay was performed in the Nexstim system through manually parcellating the brain based on the gyri. Afterwards, the errors per cortical area as well as the total number of stimulations per cortical area were recorded. Since very low numbers of stimulations could cause high ERs per cortical area, a cut-off of 25 stimulations was selected for visualization purposes. For visualization of the percentage of stimulations in each cortical, no cutoff value was used, as here we demonstrate the absolute number of stimulations occurring in each area. The vector illustrations were drawn using Adobe Illustrator (v26.0.3).

### 2.9 Statistical analysis

ERs and cortical locations of nTMS errors were exported from the Nexstim system for analysis. Statistical analysis was performed with Python 3.9, using Numpy (v1.21) (49), SciPy (v1.8) (50) and Pandas (v1.3) (51) packages. We performed independent *t-*tests for normally distributed variables and performed the Mann-Whitney U test for non-normally distributed variables. Normality was tested for using the Shapiro-Wilk test including an assessment of skewness and outliers. Significant effects were considered at p < 0.05. Fischer’s exact test was performed for categorical values when > 20% of values had a frequency < 5; otherwise, a Chi-square test was performed (52). Correlation tests were performed using the Pearson’s correlation coefficient. For multiple comparisons, statistically, significant p-values were corrected using false discovery rate (FDR) with the Benjamini–Hochberg procedure (53). Data visualization was performed using Seaborn (v0.11.2), based on Matplotlib (54).

## 3 Results

We included 60 right-handed patients (24 female, 36 male) with left-hemispheric language-eloquent brain tumors. 49 patients were being treated for WHO grade 3 and 4 gliomas (55) and 11 patients had low-grade gliomas or metastases (**Table 1**). 30 patients received the tractogram-based nTMS mapping protocol of the AF and 30 patients received the perisylvian protocol, in which the perisylvian cortex over both hemispheres was stimulated. All patients received a pre-operative AAT to assess specific components of language function. Patients were also cognitively assessed pre-operatively using the DemTect, which 2 patients did not receive due to time restraints during the pre-operative mapping. Additionally, 30 patients (50%) received a 3-7 day post-operative AAT as some patients did not receive an operation, were operated on at a different clinic, or were unwilling/unable to participate in post-operative testing.

**Table 1:** Patient demographics.

Characteristics	All patients (n=60)	Perisylvian (n=30)	Tractogram-based (n=30)	p-Value
**Gender**				.188^3^
**(female/**	24	15	9	
**males)**	36	15	21	
**Age**	49.6 ± 14.7	48.1 ± 13.5	51.0 ± 16.0	.461^1^
**WHO Grade**				.096^2^
**2**	7 (12%)	3 (10%)	4 (13%)	
**3**	21 (35%)	15 (50%)	6 (20%)	
**4**	28(47%)	11 (37%)	17 (57%)	
**Metastases**	4 (7%)	1 (3%)	3 (10%)	
**Tumor Location**				.834^2^
**Frontal**	24 (40%)	12 (40%)	12 (40%)	
**Temporal**	20 (33%)	9 (30%)	11 (3**7**%)	
**Parietal**	6 (10%)	4 (13%)	2 (7%)	
**Insular**	10 (17%)	5 (17%)	5 (17%)	
**Pre-OP AAT scores**	321.8 ± 50.0	316.2 ± 54.2	327.5 ± 20.3	.337^1^
**Post-OP AAT scores**	293.9 ± 70.6	303.6 ± 54.8	279.3 ± 90.1	.365^1^
**BAS Score**				.282^2^
**0**	48 (80%)	24 (80%)	24 (80%)	
**1**	9 (15%)	3 (10%)	6 (20%)	
**2**	1 (2%)	1 (3%)	0 (0%)	
**3**	2 (3%)	2 (7%)	0 (0%)	
**DemTect**				.925^2^
**1**	39 (65%)	19 (63%)	20 (67%)	
**2**	12 (20%)	7 (23%)	5 (17%)	
**3**	7 (12%)	3 (10%)	4 (13%)	
**Missing**	2 (3%)	1 (3%)	1 (3%)	
**Baseline ER**	14.9% ± 12.7%	14.2% ± 14.9%	15.6% ± 10.4%	.685^1^
**RMT**	32.8 ± 6.5	33.4 ± 6.4	32.2 ± 6.5	.475^1^

Values are reported for all patients and for each protocol as mean ± standard deviation (std) or n (percentage). Pre- and post-OP AAT values are out of 350. ^1^Independent t-test p-value; ^2^Fischer’s-exact test p-value; ^3^Chi-Square p-value.

### 3.1 Demographics and tumor characteristics

None of the demographic parameters assessed in **Table 1** differed significantly between the perisylvian protocol and the tractogram-based protocol. The mean patient age in the perisylvian group was 48 years old (SD = 13) and the patients who received the tractogram-based protocol had a mean age of 51 years old (SD = 16), p = .461. Additionally, 15 (50%) of the patients in the perisylvian group were women and 9 (30%) of the patients in the tractogram-based group were women (**Table 1**).

The average baseline ER for patients in the perisylvian group was 14% (SD = 15%) and in the tractogram-based group 16% (SD = 10%) (p = .685). The maximum baseline ER was 63.75%. The pre-operative AAT showed an average value of 316/350 (SD = 54) for the perisylvian group and an average value of 327/350 (SD = 20) for the tractogram-based group (p = .289), both corresponding to a BAS Score of 0 (no aphasia) (**Table 1**). The BAS scores between the groups did not differ significantly (p = .282). Three patients in the perisylvian group had a BAS Score 2 (moderate aphasia) or 3 (severe aphasia) while no patients in the tractogram-based group had moderate or severe aphasia. AAT assessments performed 3-7 days post-operatively showed an average value of 304/350 (SD = 55) for the perisylvian group and 279/350 (SD = 90) for the tractogram-based group (p = .365). The DemTect was also performed for both patient groups and showed no significant difference in distribution of results (p = .925). 63% of patients in the group with the perisylvian protocol and 67% of the patients in the group with the tractogram-based protocol showed no cognitive deficits based on the DemTect (**Table 1**).

### 3.2 Analysis of ERs

The total number of stimulations differed between the protocols. The total number of stimulations in the tractography-based protocol and the perisylvian protocol were 1,610 and 6,340, respectively. The number of errors which occurred in the tractography-based protocol was 86 and the total number of errors in the perisylvian protocol was 224. ERs were calculated per subject based on the number of errors during the stimulation divided by the total number of stimulations.

**Figure 2:**
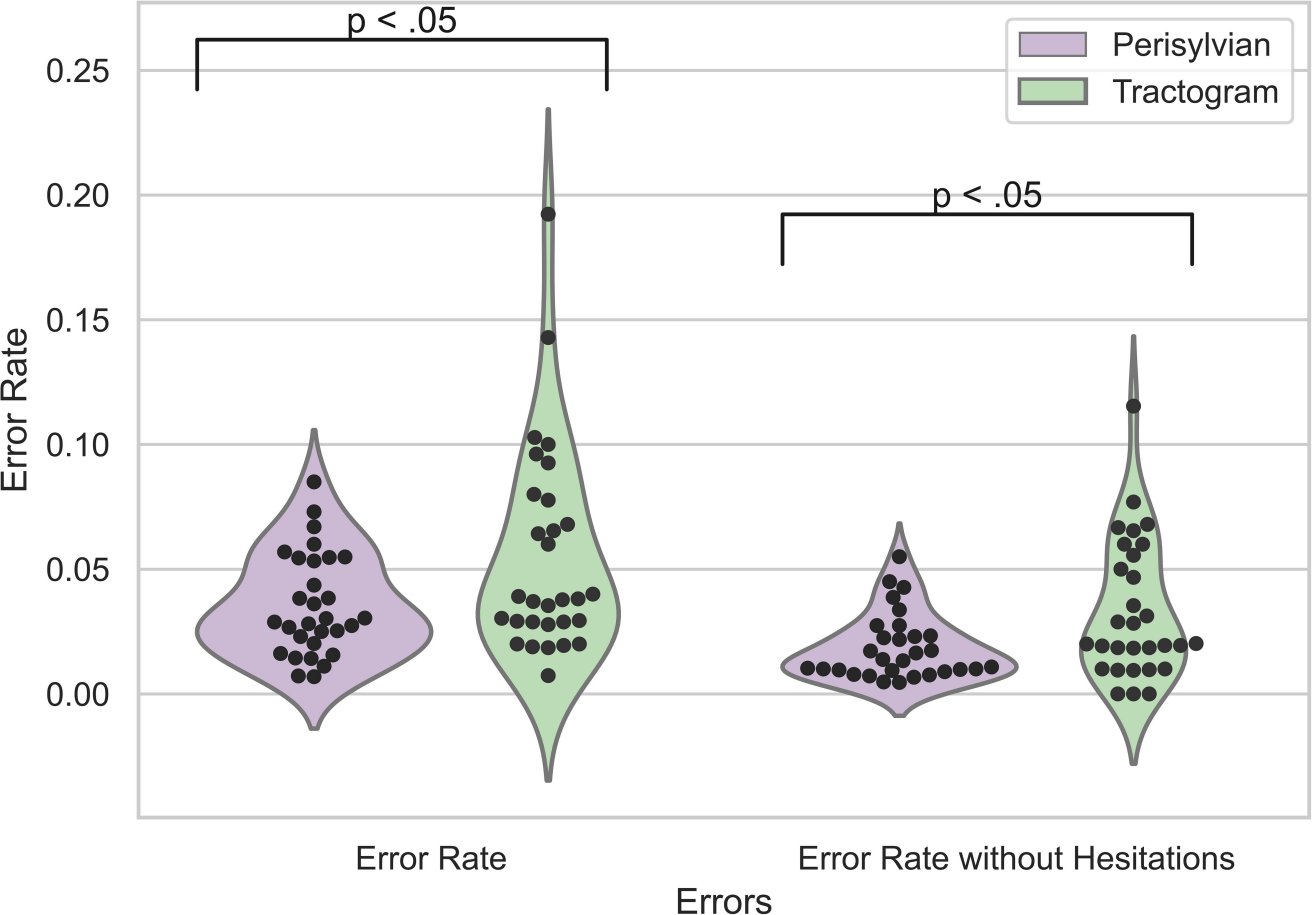
ERs of nTMS results for the perisylvian (purple) and tractogram-based (green) protocol. ER, error rate: the sum of nTMS errors divided by the total number of stimulations. The violin plot shows overall ERs as well as ERs without hesitation errors. The black dots represent the individual data points per patient.

Using a Mann-Whitney U test, the median (IQR) overall ER in the tractogram-based group was 3.8% (2.9% - 7.5%), and the median (IQR) ER in the perisylvian group was 2.9% (2.1% - 5.4%); the distributions in the two groups differed significantly (U = 316.5, n_1_ = n_2_ = 30, p = .024, two-tailed, **Figure 2**). The median (IQR) ER without hesitation errors in the tractogram-based group was 2.0% (1.2% - 5.4%) and the median (IQR) ER without hesitation errors in the perisylvian group was 1.4% (0.9% - 2.3%); the distributions in the two groups also differed significantly, (U = 302.0, n_1_ = n_2_ = 30, p = .015, two-tailed).

### 3.3 Analysis of ERs by error type

**Figure 3:**
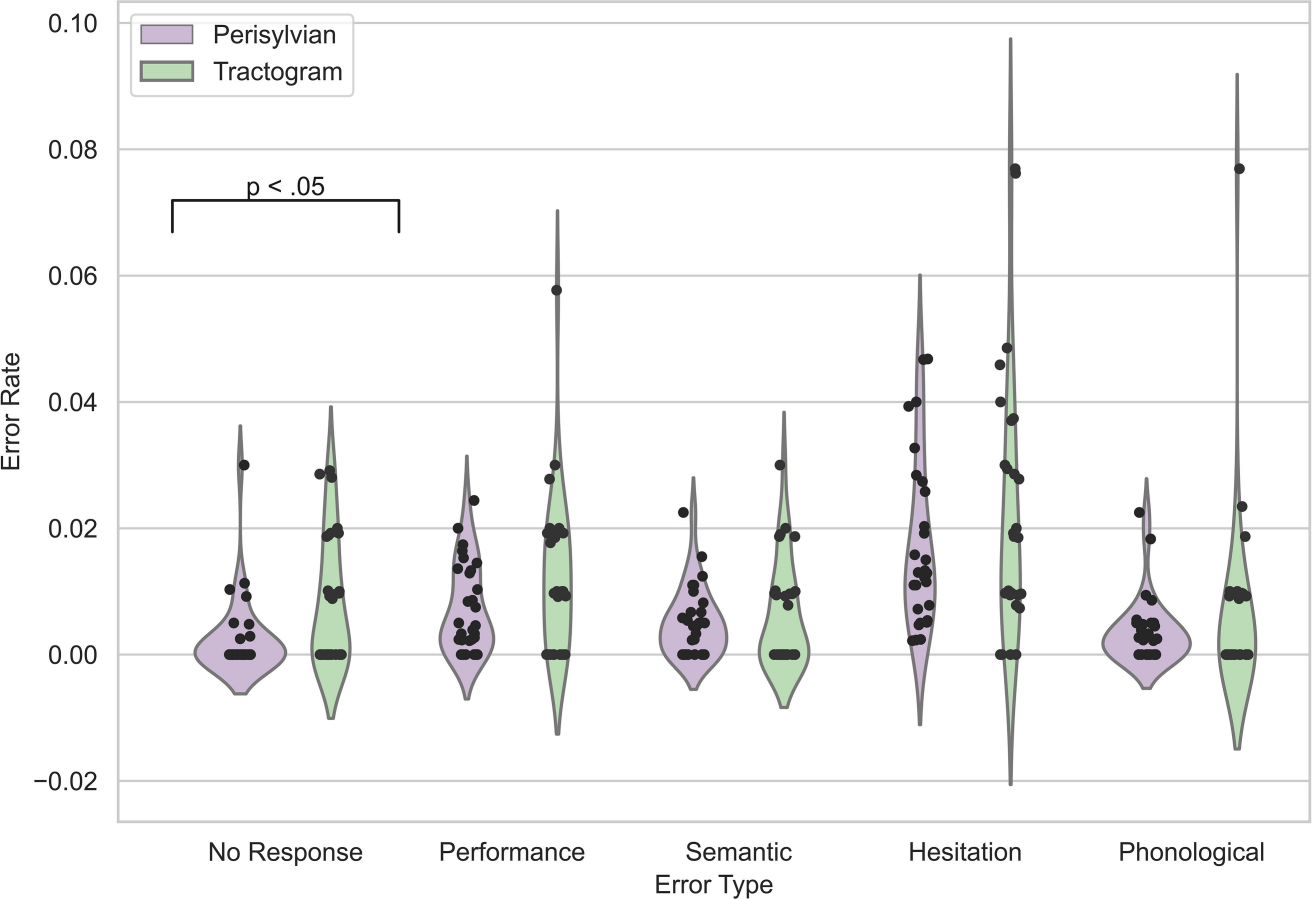
nTMS ERs grouped by recorded error types. The violin plots display ERs distributions for the perisylvian (purple) and tractogram-based (green) TMS protocols. The black dots represent the individual data points per patient.

Using a Mann-Whitney U test, the median (IQR) no-response ER of 0.4% (0.0% - 1.7%) was significantly higher in the tractogram-based protocol than in the perisylvian protocol of 0.0% (0,0% - 0,25%), p = .024). For the other error types, no significant difference between the protocols was found (**Figure 3**).

### 3.4 Correlation of baseline ER with AAT subtests

**Figure 4:**
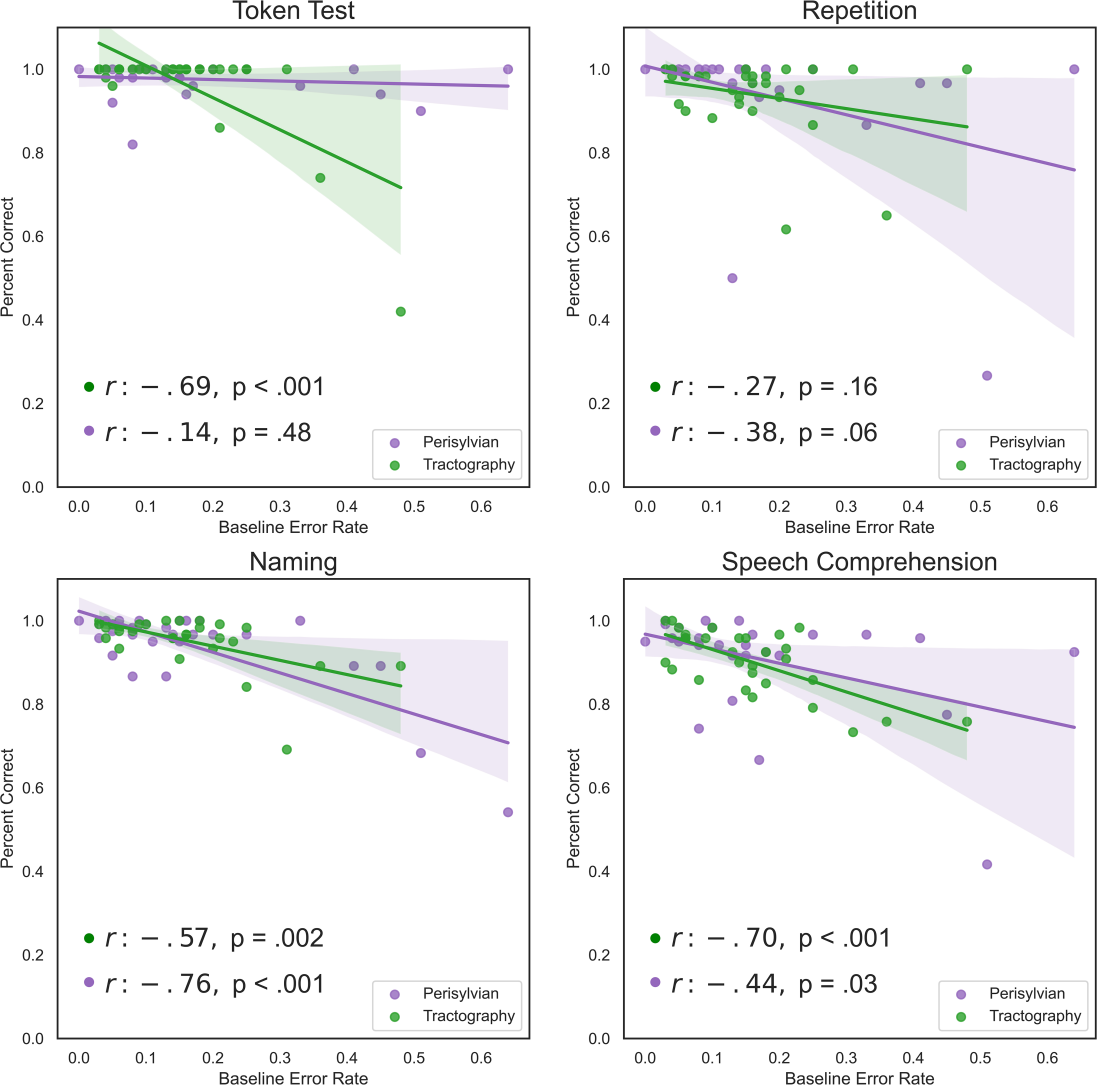
Linear regression charts between baseline ERs and the pre-operative AAT subtests. Subtests include the token test, repetition test, naming test and speech comprehension test of spoken and written language. Plotted are the percentages achieved for each test out of 100%. Regression models are visualized for perisylvian (purple) and tractogram-based (green) protocols. Legends for each subplot contain the correlation coefficient (r) and corrected p-values using the Benjamini–Hochberg procedure.

Baseline ERs showed negative correlations with some AAT subtests for both protocols (**Figure 4**). Naming for both the perisylvian (p <.001) and the tractography-based (p = .002) protocol correlated significantly with Baseline ERs, using corrected p-values. Speech comprehension also correlated negatively with Baseline ERs for both the perisylvian (p = .03) and the tractography-based (p <.001) protocol (**Figure 4**).

### 3.5 Analysis of cortical area distribution

**Figure 5:**
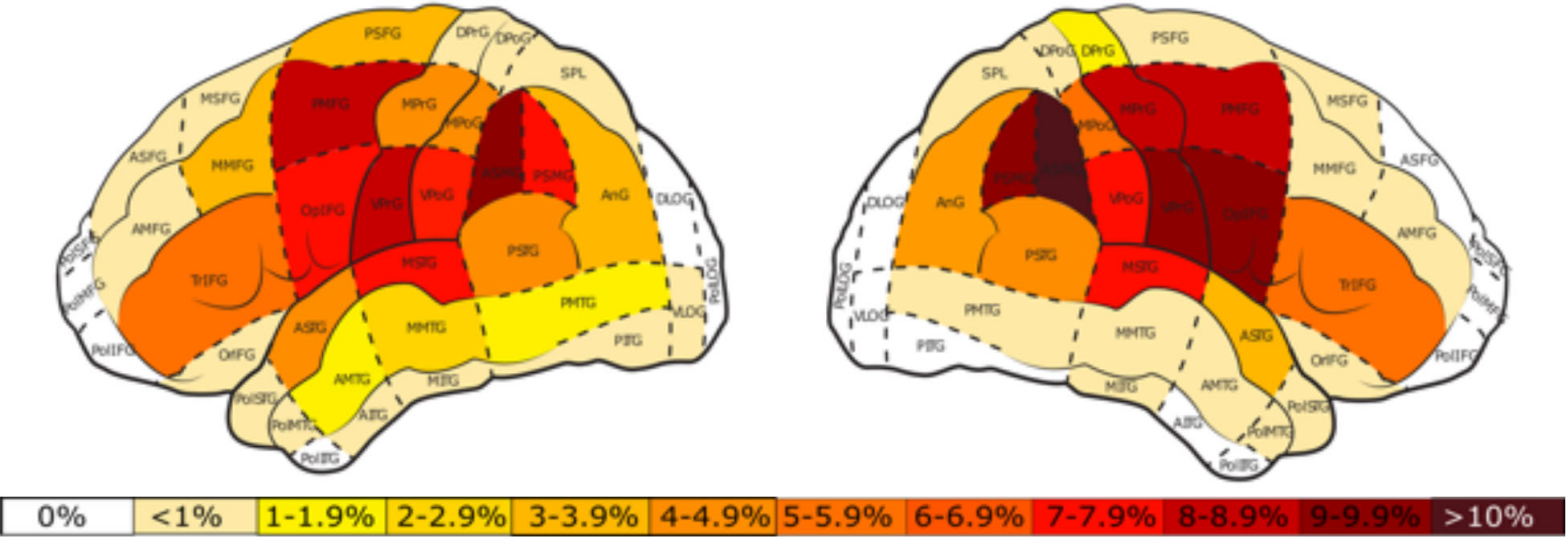
Percentage of total stimulations per cortical area for the perisylvian protocol in the left and right hemisphere. White areas represent those in which no stimulations took place. The color bar represents the percent distribution ranging from 0% (white) to >10% (dark red).

**Figure 6:**
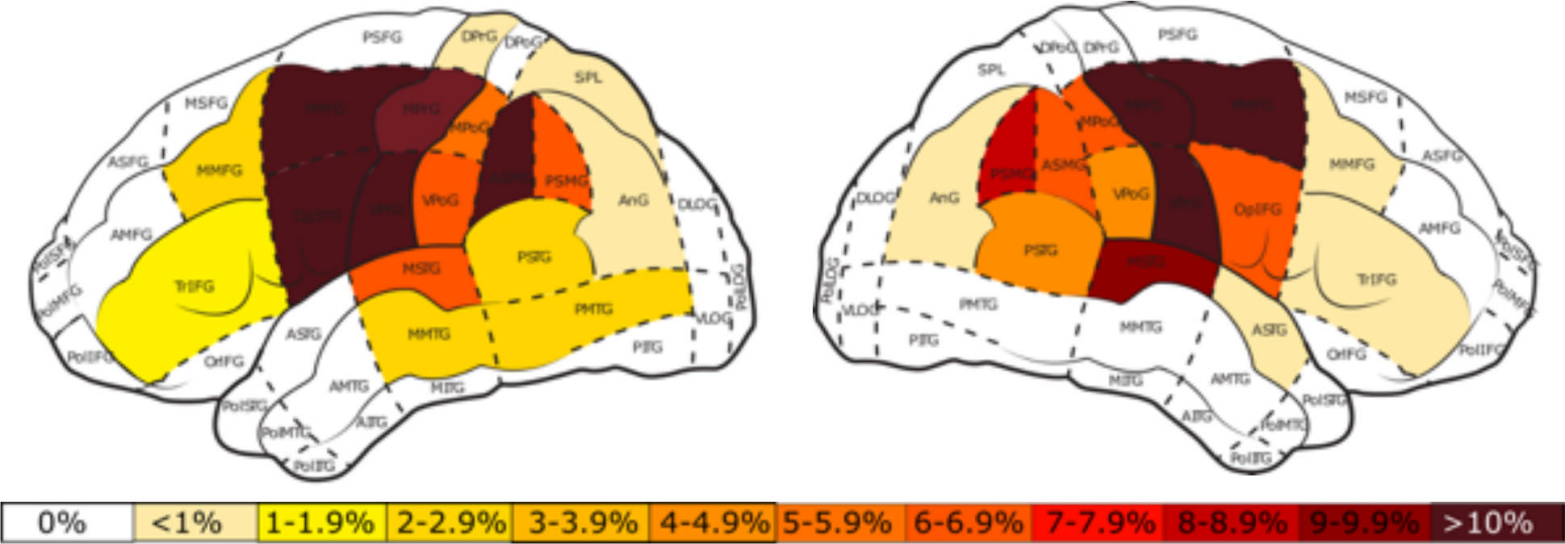
Percentage of total stimulations per cortical area for the tractogram-based protocol in the left and right hemisphere. White areas represent those in which no stimulations took place. The color bar represents the percent distribution ranging from 0% (white) to >10% (dark red).

At the conclusion of the nTMS mapping, the distribution of nTMS errors was recorded using the Corina cortical area parcellation system (47). The total number of stimulations per area for the perisylvian protocol (**Figure 5**) were distributed greater about the cortex in comparison to the tractogram-based protocol (**Figure 6**).

### 3.6 Perisylvian protocol

**Figure 7:**
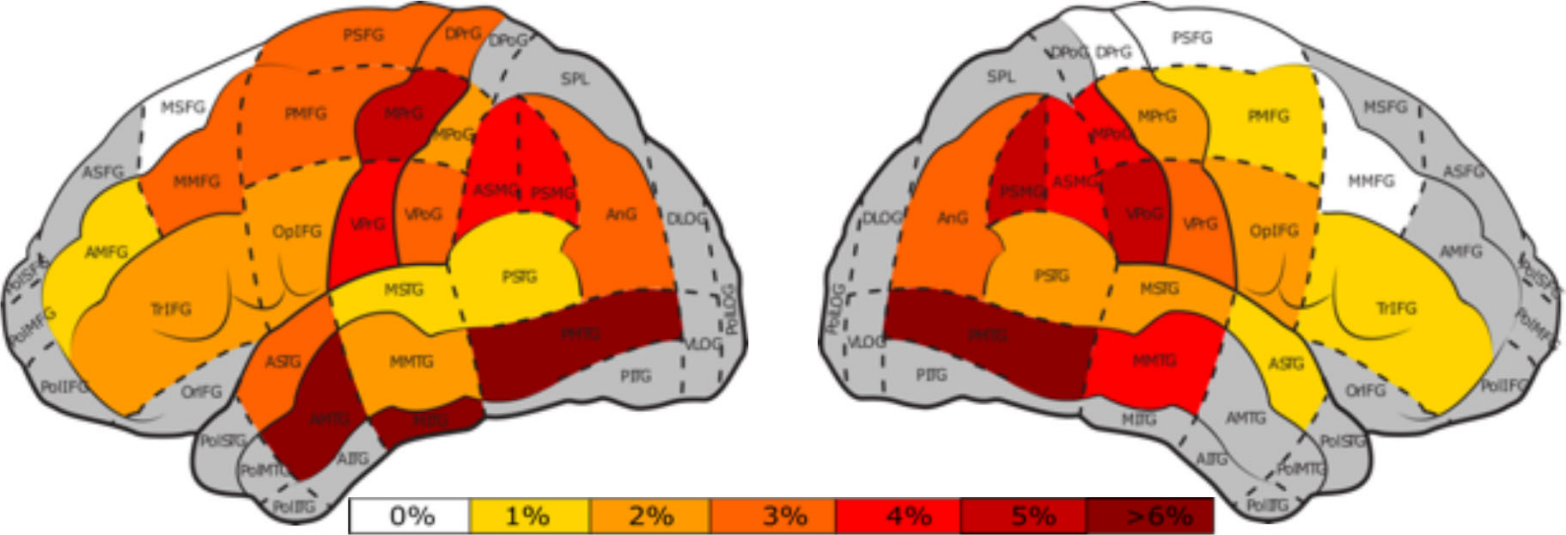
Percentage of nTMS errors in both hemispheres per cortical area (47) for the perisylvian protocol. Gray areas represent areas with less than 25 nTMS stimulations and white areas indicate areas that were stimulated without occurance of any errors. The color bar indicates the nTMS errors ranging from 0%, white, to >6%, dark red.

In the perisylvian protocol, the posterior middle temporal gyrus (pMTG) had the highest nTMS ERs in both hemispheres, left (Mean = 9.6%) and right (Mean = 9.5%). The left hemisphere also showed high ERs in the middle inferior temporal gyrus (mITG) (Mean = 6.2%) and the anterior middle temporal gyrus (aMTG) (Mean = 7.0%) (**Figure 7**). No-response ERs in the left-hemisphere were highest in the angular gyrus (anG) and the middle pre-central gyrus (mPrG). Performance ERs were most prominent in the pMTG and dorsal pre-central gyrus (dPrG). Phonological ERs were highest in the anG and middle post-central gyrus (mPoG).

### 3.7 Tractogram-based protocol

**Figure 8:**
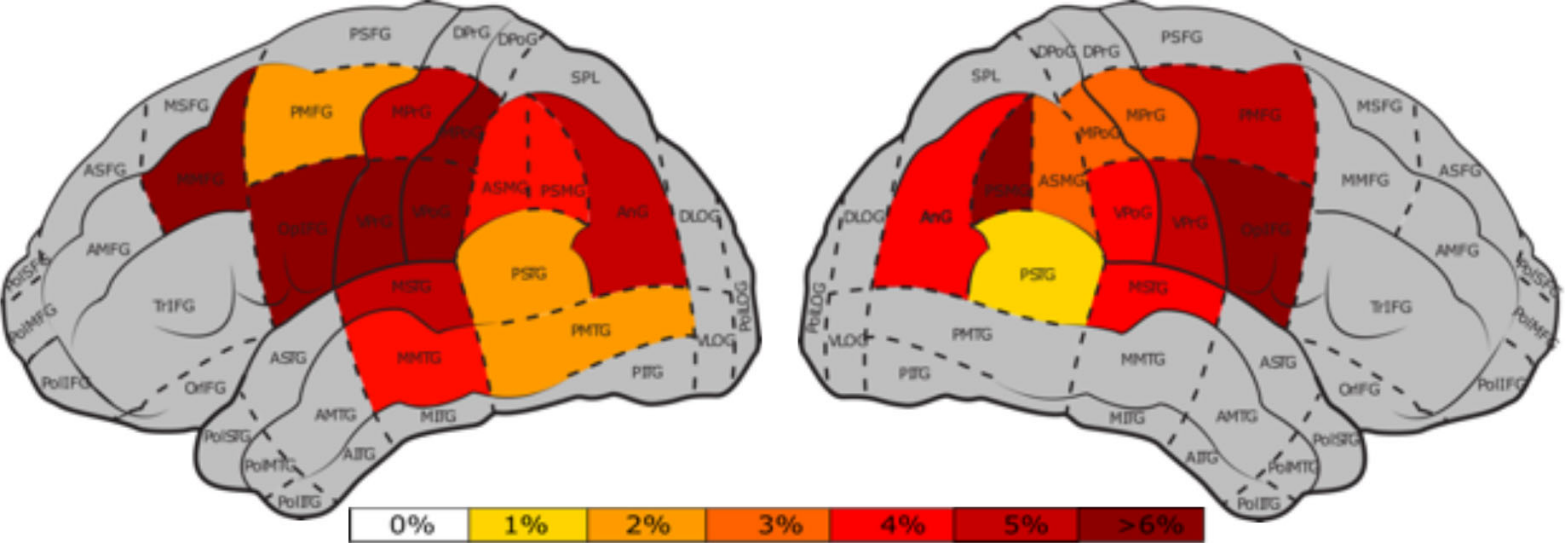
Percentage of nTMS errors per cortical area (47) for the tractogram-based protocol, for the left and right hemisphere. Gray areas represent areas with less than 25 nTMS stimulations and white areas indicate areas that were stimulated without occurance of any errors. The color bar indicates the nTMS errors ranging from 0%, white, to >6%, dark red.

The distribution of stimulation points was greater in the left hemisphere of the tractogram-based protocol than in the right hemisphere due to tumor-induced displacement of the AF. The highest ERs in the left hemisphere were found in the ventral post-central gyrus (vPoG) (Mean = 9.1%), the vPrG (Mean = 8.8%), middle middle frontal gyrus (mMFG) (Mean = 6.3%) and the opercular inferior frontal gyrus (opIFG) (M = 6.2%) (**Figure 8**). The right hemisphere showed high ERs in the opIFG (Mean = 8.9%) and in the posterior supramarginal gyrus (pSMG) (Mean = 13.2%) (**Figure 7**).

No-response ERs were most prevalent in the anterior superior temporal gyrus (aSTG), vPoG, and opIFG of the left hemisphere. Phonological errors mainly occurred in the AF’s frontal termination points of the left hemisphere, with the highest rate of phonological errors occurring in the vPrG (Mean = .82%). Additionally, all but one phonological error occurred in the left hemisphere. Performance ERs in the left-hemisphere were highest in the opIFG and vPoG. Semantic ERs in the left hemisphere, on the other hand, were highest in the pMTG (Mean = 1.25%).

### 3.8 Correlation studies of clinical and nTMS parameters

**Figure 9:**
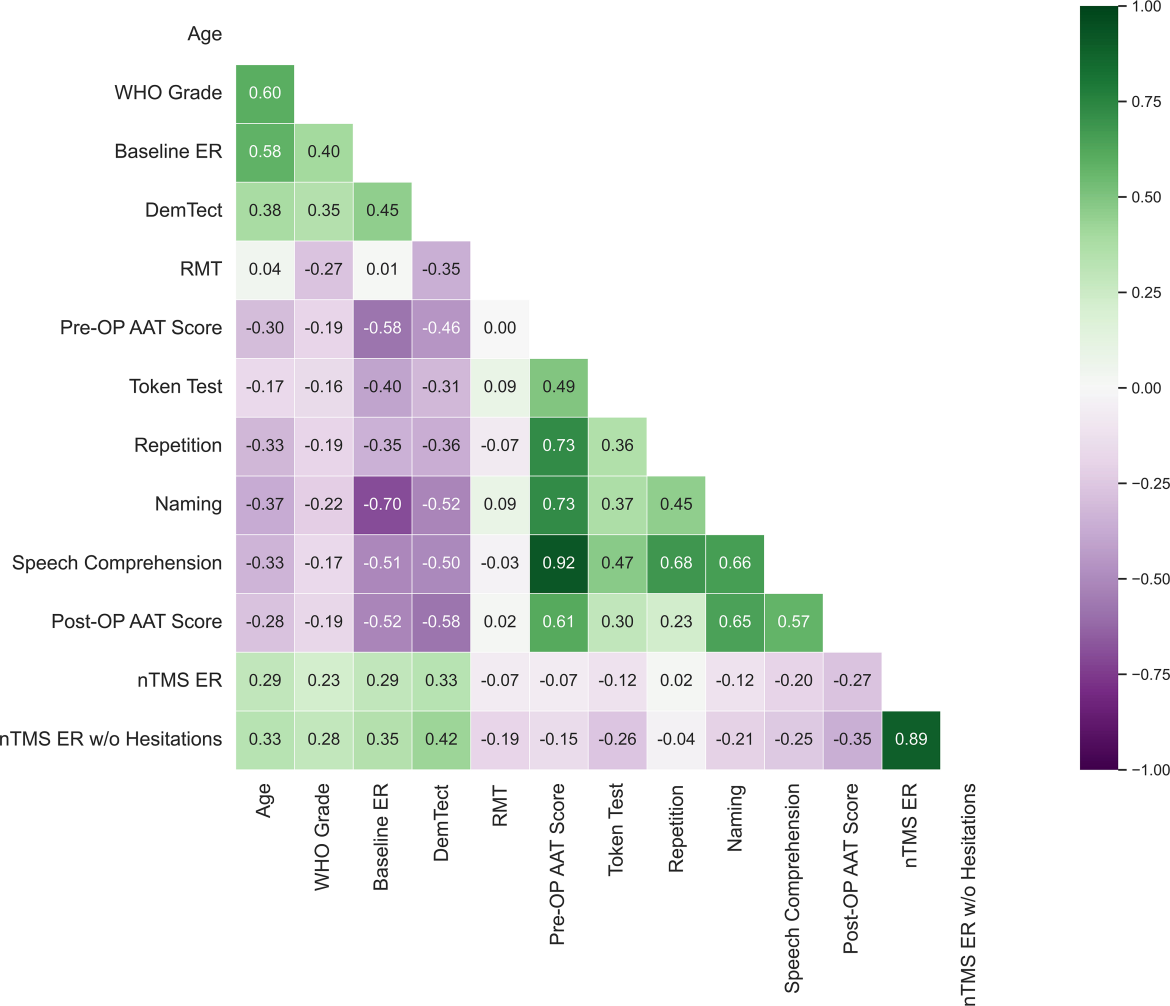
Bivariate correlation analysis between clinical and nTMS parameters. Each number represents the Pearson’s correlation coefficient between listed clinical parameters. Pre-OP AAT scores were collected on the day of the nTMS mapping and post-operative AAT scores were collected 3-7d post-operatively. Token test, repetition, naming and speech comprehension represent the sub-tests of the AAT taken pre-operatively.

Correlations studies were performed to assess the linear correlation between different clinical and nTMS parameters. Using corrected p-values, nTMS ERs correlated significantly with the DemTect-Test (p = .021) and age (p = .042) but not with WHO grade (p = .121), pre-operative (p = .676) or post-operative AAT scores (p=.061). nTMS ERs without hesitations also did not correlate significantly with pre-operative (p = .309) or post-operative (p = .083) AAT scores (**Figure 9**).

Baseline ERs correlated significantly with nTMS ER’s (p = .043) (**Figure 9**) and with nTMS ERs without hesitations (p = .014), ER of no-response errors (p = .006), and ER of performance errors, (p = .005). However, baseline ERs did not correlate significantly with the percentage of semantic, hesitation, or phonological errors. Baseline ERs showed greater correlations with the tested clinical parameters than did nTMS ER or nTMS ERs without hesitations. A Pearson correlation coefficient showed a significant positive correlation between Baseline ERs and WHO tumor grades (p = .004), DemTect-Test (p <.001), and a significant negative correlation with post-operative AAT scores (p = .004) (**Figure 4**).

## 4 Discussion

In the present study, we established a tractogram-based nTMS language mapping protocol with AF-targeted stimulation points. Evaluating functional language areas provides meaningful information for neurosurgical preoperative planning. Though direct electrical stimulation (DES) is still considered the gold standard for mapping language function, it is invasive, performed intra-operatively during awake surgeries, and is not feasible for all patients (4). Thus, nTMS language mapping offers a potential means towards non-invasive, pre-operative mapping of language areas, but its specificity rates remain low for its reliable clinical use (56). This study attempted to address the need for more reliable nTMS by standardizing nTMS targets and mapping endings of language-relevant fiber bundles like the AF.

### 4.1 Tractography

Using the AF’s cortical endpoints as stimulation markers acknowledges the difference in gyral anatomy between patients, especially in those with anatomy-disrupting pathologies. Additionally, it allows for a targeted stimulation of individual fiber bundles and enables more specific analysis of functionality in relation to the targeted fiber bundle. This method demonstrates an alternative approach to current nTMS language mapping and was achieved using CSD-based probabilistic tractography with MRTrix3 (26).

CSD-based probabilistic tractography provides a more accurate delineation of fiber bundles and can accommodate crossing fibers compared to tensor-based deterministic tractography (57). Even so, the latter approach remains the predominant method implemented in neurosurgical practice (58). This predominance is also reflected in the use of tensor-based deterministic tractography by third-party providers e.g. Stryker (Kalamazoo, MI, USA) or Brainlab (Brainlab AG, Munich, Germany), which provide clinical softwares for pre-operative planning and tractography (58). Medtronic (Minneapolis, MN, USA) seems to be currently developing an implementation that should enable tractography algorithms based on CSD. In order to perform probabilistic CSD-based tractography for this study, MRtrix3 was used to generate fiber bundles for the tractogram-based protocol (44) and results were uploaded into Brainlab for easy clinical access using Karawun (45).

### 4.2 nTMS

Both protocols were well-tolerated by patients and no complications, such as seizures, occurred. This finding supports the previously documented overall safety and tolerability of nTMS (59). nTMS ERs and nTMS ERs without hesitations did not correlate significantly with pre-operative or post-operative AAT scores, which supports previous studies that pre-existing language disturbances do not correlate significantly with nTMS mapping outcomes (2). Additionally, naming and speech comprehension AAT subtests had moderately significant correlations with baseline ER for both protocols (**Figure 4**). There was a significant correlation between baseline ERs and nTMS ERs, though this correlation was weak (r = .29). In total, baseline ERs rather than overall AAT scores would be preferred in determining cut-off points for mappings and reducing confounding effects of aphasia on nTMS outcomes.

In the tractogram-based protocol, overall ERs and no-response ERs were significantly higher than in the perisylvian protocol, pointing towards increased nTMS responsiveness with the tractogram-based protocol. Even so, the other error types did not differ significantly between the two groups and the generally low ERs during mappings reinforce the difficulty of using nTMS as a reliable method to identify language areas. Studies have shown that implementing different language tasks like action naming (60) or modifying the protocol to increase stimulation frequency (61) may contribute to a more effective nTMS mapping.

Language function is primarily associated with the left-hemisphere in right-handed patients. Interestingly, no significant difference in ERs was observed between the left and right hemisphere for either protocol. This finding supports nTMS findings demonstrating that tumor-induced plasticity may cause increased recruitment of the language network to the right hemisphere (46). Plasticity enables dynamic redistribution of neuronal networks by limiting the expression of lesion-related impairment and assisting in post-surgical recovery (62, 63). However, upon examining the distribution of error types, phonological errors were especially rare in the right hemisphere, supporting left-hemispheric dominance for phonological function (64) and its persistent dominance even in tumor patients.

### 4.3 Targeted nTMS with the AF

Establishing anatomical correlates for cortical termination of fiber bundles is challenging and shows interindividual differences (13). Our understanding of the AF’s anatomy and projection arises from 65, which suggests in addition to a long segment of the AF, there exist two indirect pathays: the anterior and the posterior pathways (65). These connections are reflected in a variety of frontal, parietal, and temporal terminations of the AF (13). A systematic review by 66 of the AF’s anatomy showed main fibers of the indirect anterior pathway running from the posterior opIF/vPrG to the supramarginal gyrus (SMG) and the indirect posterior pathway running from the pMTG to the anG (66). The AF was selected for this study based on its strong involvement in language as the main tract of the dorsal stream (13). DES stimulation of the AF has resulted in speech arrests in both the frontal and parietal lobe (67) and injury to the tract has been documented as the most common reason for post-operative aphasia (18).

ERs based on error types were analyzed between protocols and showed significantly higher no-response ERs in the tractogram-based protocol. In the tractogram-based protocol, the opIFG, aSTG and the vPoG in the left-hemisphere of the tractogram-based protocol showed the highest no-response ERs, in line with previous studies demonstrating a prominence of no-response errors in the m/vPrG, and opIFG (47). Conversely, high no-reponse ERs in the perisylvian protocol mainly occurred in the anG. The anG is an area of the parietal lobe involved in semantic retrieval and in previous TMS experiments was shown to disrupt thematic judgements (68). Notably, no-response errors are difficult to assess since they are inaudible and the functional cause of their occurrence (disruption in language comprehension vs. production) is difficult to discern if no lead-in phrase is used (47).

The tractogram-based protocol in the left hemisphere showed the highest phonological ERs in the vPrG. In the right hemisphere, the highest ERs were found in the mPrG as well, both regions of the pre-motor cortex. However, due to its role in motor control, stimulations to regions of the v/mPrG may induce phonological disturbances through dysarthria (56). Performance ERs in the tractogram-based protocol were highest in the opIFG, consistent with its role in speech processing (14). Highest semantic ERs in the left-hemisphere for the tractogram-based group were found in the pMTG, a region implicated in language comprehension (69), syntactic information processing, and early semantic retrieval (68). Additionally, nTMS language findings have also shown pMTG to be a prominent cortical region involved in semantic paraphasias (47).

In the tractogram-based protocol, the highest overall ERs were found in the left hemisphere around the vPoG, though this area is primarily known for its role in somatosensory functioning and not language. Even though studies have also shown high ERs of the vPoG in nTMS language mappings, these did not coincide with findings from DES (70). An explanation for this may be the displacement of AF streamlines by the tumor, causing streamlines to project into different cortical regions.

Additional high overall ERs in the tractogram-based protocol mainly occured in the frontal lobe areas. In the left hemisphere, high ERs were found in the vPrG, an area coinciding with the indirect anterior segment of the AF (66) and consistent with other nTMS studies showing high nTMS ERs in the vPrG (56). Additionally, in both hemispheres, the tractogram-based protocol showed high ERs in the opIFG, an area of the frontal lobe traditionally implicated in speech production and constituting the terminal endings of the AF’s anterior indirect segment (13, 66). This high nTMS ER in the opIFG aligns with findings from language mapping studies in brain tumor patients (3, 5, 23), though it is interesting that the perisylvian protocol showed comparitively low ERs in the opIFG. This may be due to less targeted stimulations or defining regions like opIFG on a large cortical area.

While regions of the frontal lobe showed overall higher ERs in the tractogram-based protocol, the perisylvian protocol mainly demonstrated high ERs in temporo-parietal cortical areas. Interestingly, in the perisylvian protocol, the highest ERs for both hemispheres were found in the pMTG, the site of ventral terminations of the AF’s indirect posterior pathway (71, 72). Although this region has not been typically associated with the AF, tractography dissection studies have shown extensive AF cortical terminations in the pMTG (17, 68). Additionally, a previous nTMS studies stimulating the pMTG showed its involvement in semantic retrieval (68) and its prominence in relation to semantic errors (47). High ERs in the perisylvian protocol were also found in the inferior parietal lobe in both hemispheres, specifically the pSMG coinciding with the posterior terminations of the AF’s anterior segment (66). Previous nTMS findings have also shown correlation with the SMG to high ERs during language mappings (23).

The summarized differences in error distributions between protocols demonstrated higher frontal ERs in the tractogram-based protocol and higher temporo-parietal ERs in the perisylvian protocol. This effect may have been strengthened by a low number of temporo-parietal stimulation points in the tractogram-based protocol (two stimulation points in the temporo-parietal region per patient). In contrast, the tractogram-based protocol stimulated three points in the frontal lobe per patient and showed higher ERs in the aforementioned areas like the opIFG and the vPrG. Recent studies involving brain tumor patients demonstrated correlations of the posterior perisylvian area with existing language deficits (29) and injury to the temporo-parietal areas with post-operative deficits (18). It seems these temporo-parietal regions of the AF may exhibit higher significance than once thought. Although more research is required on this, these results may be clinically useful and suggest that these areas should be given more consideration in future nTMS studies.

### 4.4 Future outlook

Although the AF has been the dominant subject of language connectivity for the past 150 years (13), other relevant white matter tracts involved in language function may be applied to the protocol for future nTMS studies to analyse their effect on language function and provide a more comprehensive mapping area. For instance, the ILF, which connects occipital and temporal lobes, has shown relevance in visual semantic memory and object recognition, as well as processing visual cues (73). The IFOF connects occipital and frontal lobes and has shown to be also invovled in semantic processing and vital for visual switching tasks (74). The UF, an anterior tract connecting the frontal and temporal lobe, has shown involvement in semantic retrieval and memory function (75), though whether it remains essential for language is speculative (76). The extreme capsule, linking the anterior inferior frontal gyrus (aIFG) to the temporal cortex is also involved in semantic processing including evidence of phonological working memory (13). Additionally, the MdLF connects the superior temporal gyrus (STG) to the anG and postulations in its role in phonetic and auditory processing have been made (77). These fiber bundles implicated in language function could be future targets for a tractography-based mapping protocol. This would allow for functionally tailored mapping protocols according to the targeted tract. Additionally, we did not correlate the nTMS points in this study with respect to DES intraoperative stimulations. This would be beneficial in future studies to better distinguish false-positive points during the mapping.

## 5 Limitations

Though the means of tested demographic parameters did not differ significantly between the two groups (**Table 1**), the sequential allocation of patients through non-blinding may have introduced bias. As this study was used to demonstrate proof-of-concept, future and more extensive studies should consider further measures to ensure bias reduction. The heterogeneity of the patient population could introduce confounding factors which affect nTMS outcomes. Several parameters correlated with one another, such as nTMS ERs and baseline ERs, though this correlation was weak. Nevertheless, ensuring a sensible cut-off value for mapping language function with nTMS is vital for accurate comparison. A lower baseline ER for exclusion may prove reasonable to reduce confounding effects of language deficits on nTMS outcomes. The comparison between the two protocols has several challenges, as the perisylvian protocol uses sequential stimulation and the tractography-based protocol stimulated one point continuously before moving onto the next. Additionally, the number of stimulations differed between the two groups, which could also influence the TMS results. Additionally, nTMS error analysis is user-dependent and differs between research sites and protocols (30). Additionally, pain from stimulation especially around the temporal area limits the validity of errors and not all cortical areas are reachable during stimulation. Stimulation of facial muscles during nTMS may cause dysarthria, resulting in categorization as a performance error.

Furthermore, a central limitation to the tractogram-based protocol is that its stimulation points are AF-ending specific, while in turn disregarding other potentially essential language areas. This increases the likelihood that these language areas are overlooked during stimulation. Incorporating additional fiber bundles may be beneficial for creating a more comprehensive overview of major cortical tract endings and assessing whether these are truly essential for language. The incorporation of more language-related tracts also would benefit those patients, whose tumors lie outside the area of the AF.

Tractography suffers from various limitations, which problematizes its usage (78). Limitations of tractography include an inability to distinguish between afferent and efferent streamlines (79). Additionally, false positive (80) and false negative (81) streamlines pose limitations and are challenging to assess. Performing ROI-based tractography is user-dependent, and using anatomical landmarks as ROIs aids in standardization (41). However, tractography results vary between and within protocols, therefore improving standardization and reproducibility of fiber bundles is important (43). Therefore, automated tractography algorithms like TractSeg (82) may be used for increased standardization of tractography methods.

## 6 Conclusion

This study demonstrates the feasibility of using a protocol stimulating mapped endings of the AF during language nTMS, allowing standardized and targeted stimulation. In comparison to the perisylvian protocol, results showed evidence of increased responsiveness to nTMS language mappings when using the targeted protocol. Even so, the difficulties of using nTMS language mapping as a reliable clinical tool remain pertinent. This method could also be applied to further fiber bundles relevant to the structural language network to make nTMS language mapping more reliable and to further investigate the functional role of these fiber bundles.

## Data availability statement

The datasets presented in this article are not readily available due to patient privacy regulations, patient data cannot be distributed or shared. Requests to access the datasets should be directed to KR, klara.reisch@charite.de.

## Ethics statement

The studies involving human participants were reviewed and approved by the Charité’s Ethics Committee (EA1/005/20). The patients/participants provided their written informed consent to participate in this study.

## Author contributions

KR, LF and TP designed the study. KR and LF processed the data, performed all analyses, and wrote the first draft of the manuscript. FB, MT, and HS assisted in collecting data. PV contributed to the project management through discussions. All authors contributed to the article and approved the submitted version.

## Glossary

**Table:**

AAT	Aachen aphasia test
AF	arcuate fasciculus
ANTs	advanced normalization tools
anG	angular gyrus
aSMG	anterior supramarginal gyrus
aSTG	anterior superior temporal gyrus
BAS	Berlin aphasia score
CSD	constrained spherical deconvolution
CST	cortical spinal tract
DemTect	demenz detection test
DES	direct electrical stimulation
dMRI	diffusion magnetic resonance imaging
dPoG	dorsal post-central gyrus
dPrG	dorsal pre-central gyrus
DTI	diffusion tensor imaging
DWI	diffusion-weighted imaging
ER	error rate
FDI	first dorsal interosseus
fODF	fiber orientation distribution functions
FSL	FMRIB Software Library
iFOD2	second order integration over fiber orientation distributions
IFOF	inferior fronto-occipital fasciculus
ILF	inferior longitudinal fasciculus
IPI	inter-picture interval
MdLF	middle longitudinal fasciculus
mMFG	middle middle frontal gyrus
mMTG	middle middle temporal gyrus
mPoG	middle post-central gyrus
mPrG	middle pre-central gyrus
mSFG	middle superior frontal gyrus
mSTG	middle superior temporal gyrus
opIFG	opercular inferior frontal gyrus
orIFG	orbital part of the inferior frontal gyrus
pMFG	posterior middle frontal gyrus
pMTG	posterior middle temporal gyrus
polIFG	polar inferior frontal gyrus
polMFG	polar middle frontal gyrus;
polMTG	polar middle temporal gyrus
polSFG	polar superior frontal gyrus
polSTG	polar superior temporal gyrus
pSFG	posterior superior frontal gyrus
pSMG	posterior supramarginal gyrus
pSTG	posterior superior temporal gyrus
PTI	picture-totrigger interval
RMT	resting motor threshold
ROI	region of intest
SPL	superior parietal lobe
trIFG	triangular inferior frontal gyrus
TMS	transcranial magnetic stimulation
UF	uncinate fasciculus
vPoG	ventral post-central gyrus
vPrG	ventral pre-central gyrus
